# Effect of Fiber Reinforcement Type on the Performance of Large Posterior Restorations: A Review of In Vitro Studies

**DOI:** 10.3390/polym13213682

**Published:** 2021-10-26

**Authors:** Enas Mangoush, Sufyan Garoushi, Lippo Lassila, Pekka K. Vallittu, Eija Säilynoja

**Affiliations:** 1 Turku Clinical Biomaterial Center (TCBC), Institute of Dentistry, University of Turku, 20100 Turku, Finland; sufgar@utu.fi (S.G.); liplas@utu.fi (L.L.); pekval@utu.fi (P.K.V.); 2 Department of Biomaterials Science, Institute of Dentistry, University of Turku, 20100 Turku, Finland; eija.sailynoja@gc.dental; 3 City of Turku Welfare Division, Oral Health Care, 20100 Turku, Finland; 4 Reseach and Development and Production Department, Stick Tech Ltd.—Member of GC Group, 20100 Turku, Finland

**Keywords:** fiber-reinforced composite restoration, polyethylene fiber, glass fiber, fracture resistance, microleakage

## Abstract

To reinforce extensively prepared cavities, different types of fiber reinforcement are utilized. Polyethylene and glass fibers are the most commonly used fibers in that purpose; each type has its own advantages over the other type. Therefore, the aim of this study is to review the literature to evaluate and compare the influence of different fiber reinforcement types on the performance of posterior large composite restorations. Two independent authors performed a comprehensive literature search using MEDLINE/PubMed, Google Scholar, and a manual search for cross references until July 2021. Authors selected only studies that contain comparisons between glass (continuous or short) and polyethylene (woven) fiber-reinforced composites (FRCs) in posterior cavities of human teeth, and that report the effect of fiber inclusion on fracture resistance, microleakage, and marginal adaptation of restorations. A number of 2711 potentially relevant articles were obtained from the electronic search. After extensive assessment, 2696 articles were ineligible to be included in the review, and only 15 articles met the inclusion criteria. Four out of nine studies, which tested the fracture resistance of FRC restorations, revealed similar performance of the glass and polyethylene fibers. The rest of the studies (*n* = 5) revealed statistically significant differences between the two types of fiber reinforcement, with the majority showed superior reinforcement of glass fiber. Moreover, the reviewed studies revealed that, using fibers within the composite restorations would reduce the microleakage and improve the marginal adaptation of the restoration regardless of the fiber type. FRCs tend to strengthen the restorations of structurally compromised teeth and improve their performance compared to plain composite restorations.

## 1. Introduction

Extensive cavity preparation is one of the major contributing factors of tooth fragility, which could result in the partial or complete fracture of cusps or roots of posterior teeth [1]. For instance, preparation of MOD cavities causes up to 54% reduction in the tooth fracture strength compared to non-prepared teeth [2].

Innovative treatment solutions based on new improved materials are continuously evolving to restore the function and preserve the remaining tooth structure, with resin composite being a prime example. Resin composite can bond to tooth structure, which means, theoretically, it has the ability to regain the lost fracture resistance, and to strengthen the tooth by providing an internal splint. However, the reinforcing effect of direct composite fillings when applied alone in large cavities is highly debated [3]. The structural performance of resin composite fundamentally depends on the damage tolerance limit and the fatigue resistance of the material [4]. The basic problems of the composite restorations are insufficient toughness and increased contraction, as well as polymerization shrinkage stress [3]. The polymerization shrinkage increases as the cavity depth increases, because of the greater cantilever effect and the greater volume of restorative materials, which is generally seen in big cavities and root-canal-treated teeth, as the pulpal floor is lost [5]. The increased shrinkage stress results in marginal breakage, microleakage, and secondary caries [5]. 

The advent of fiber reinforcement has expanded the potential applications of composite restorations in restorative dentistry, as they internally strengthen the restorations and reduce the occurrence of fractures [6]. The most commonly used fiber-reinforced composites (FRCs) types are polyethylene ribbon and glass FRCs. Both types have been revealed to play an important role in increasing the fracture strength of restorations of endodontically treated and non-endodontically treated teeth [4,7,8], and improving the microleakage and marginal integrity of the restorations [9,10,11]. 

During the past two decades, a leno woven ultra-high molecular weight (LWUHMW) polyethylene fiber ribbon has been used to reinforce cavities [7]. As an example of the commercially used polyethylene fibers is the non-impregnated fiber ribbon (Ribbond), it is treated with cold gas plasma to enhance their chemical bond to the applied restorative materials [12]. However, some literature findings have highlighted the inadequate adhesion between polyethylene fibers and polymer matrix [13,14]. They are placed either under the composite restoration or over it in a prepared groove [7,15], or circumferentially inside the axial walls [16]. Polyethylene fibers act as a layer to absorb stresses, and to internally splint the tooth and reinforce the composite in more than one direction [17].

Continues (uni and bi-directional) glass fiber-reinforced composites have also demonstrated their ability to improve the fracture strength and to stop crack propagation in composite restorations [18,19,20]. Moreover, glass FRCs are capable of improving marginal integrity and microleakage when used as a resin composite substructure [19]. An example of the continues FRC is the pre-impregnated E-glass FRC (everStick). They have a semi-interpenetrating polymer network structure (semi-IPN) which, based on the ability of the polymer matrix to dissolve partially in the bonding resin, clinically leads to superior bonding properties [21]. 

Short fiber-reinforced composites (SFRC) have also been widely used as bulk base in high stress-bearing areas to reinforce the large restorations and to mimic the stress absorbing properties of dentine [22]. The resin matrix of the SFRC contains, in addition to inorganic particulate fillers, short and randomly oriented glass fibers that provide a three-directional reinforcement. Fibers in the SFRC showed the ability to re-direct and stop crack propagation within the composite [21]. 

With these varieties of fiber reinforcement types, the question arises as to whether polyethylene and glass FRCs similarly reinforce the cavities, or if one has a preferable performance over the other. There are limited scientific data to influence clinicians’ decisions when deciding which material to choose; therefore, the aim of this study is to conduct a review to evaluate and compare the reinforcing effect of polyethylene and glass FRCs on the performance of posterior large-composite restorations.

## 2. Materials and Methods

This systemic review was achieved following the guidelines of PRISMA 2020 statement [23].

### 2.1. Search Strategy and Data Collection Process 

A comprehensive electronic literature search was conducted up until July 2021 via MEDLINE/PubMed and Google Scholar; in addition, a manual search of references of the selected studies was performed to analyze all the available potential articles. The following terms were used as search keywords: fiber-reinforced composite in dentistry, polyethylene and/or glass fiber-reinforced composite, polyethylene and short fiber-reinforced composite, fiber cavity reinforcement, woven and unidirectional fibers, Ribbond fibers, everStick fibers, everStick NET, everStick C&B, everX Posterior, everX Flow, Ribbond and everStick, Ribbond and everX Posterior, Ribbond and everX Flow, fracture resistance of fiber-reinforced composite.

### 2.2. Eligibility Criteria 

Eligible studies for inclusion in this review are full-text studies that tested the fracture resistance, microleakage and marginal adaptation of composite restorations reinforced by both polyethylene and glass FRCs. Materials should be used only within the tooth cavity as a substructure or core build-up or filling material, but not in the root. Studies should be published in English language peer-reviewed journals; the search terms were included in either the title or abstract. Included Studies used only extracted human molars or premolars to test the materials. Articles that do not contain direct comparison, unpublished studies, personal communications, background information, and conference abstracts were excluded.

### 2.3. Selection Process

Full texts of the potentially relevant articles, according to the inclusion criteria were gained and potential duplicates were carefully read before exclusion. Different reasons for exclusion were agreed between authors. The included articles were evaluated for the presence of the following factors: the two types of fibers were used in a comparable manner and tested under the same conditions.

### 2.4. Data Synthesis

The included articles were carefully read, and data of interest were extracted and reported in Microsoft Word files. For each single article, the authors’ names, year of publication, evaluated parameters, type of control group, type and commercial name of evaluated fiber-reinforced composite, FRCs application technique, the main results and conclusions were reported. 

### 2.5. Quality Assessment

Two authors (EM, SG) independently assessed the risk of bias of the included studies. This was undertaken according to two previous systemic reviews [24,25]. The following eight parameters were used to evaluate the risk of bias: presence of control group; samples preparation standardization; samples randomization; samples preparation by single operator; materials used according to manufacturer’s instruction; blindness of the operator during testing; clarification of calculation of sample size; failure mode evaluation (this one only applied for fracture resistance studies). If a parameter was mentioned in the study, it was recorded as (YES), while if it was not mentioned it was recorded as (NO). According to number of (YES) answers, risk of bias was determined as high (1–3 YES), medium (4–6 YES) or low risk (7–8 YES).

## 3. Results

A total of 2711 relevant articles were recognized and screened for title and abstract evaluation. After assessment, 2696 were removed because they did not meet the eligibility criteria or due to duplication. Fifteen full-text articles were evaluated, and two articles were excluded due to using the tested material as an endocrown or extended it to the roots. Two more articles were found by manual search and cross references [26,27] producing a number of 15 full-text comparative articles, that entirely met the inclusion criteria [4,8,9,10,11,26,27,28,29,30,31,32,33,34,35].

**Figure 1:**
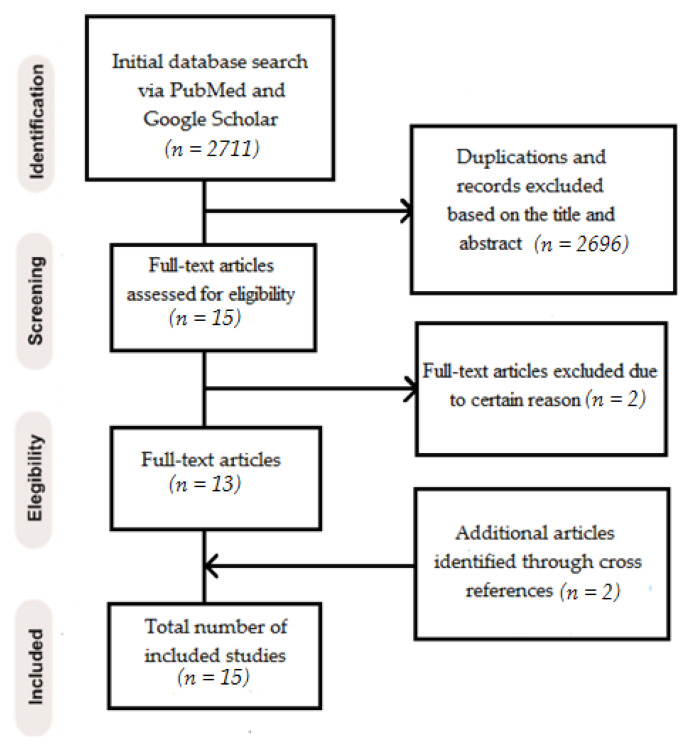
Screening and selection process in PRISMA flow diagram.

Figure 1 shows a PRISMA flow diagram for the screening and selection process. Within the 15 included studies, nine published articles compared the fracture resistance of restorations reinforced by glass vs. polyethylene FRCs applied within extracted human teeth [4,8,26,27,28,29,30,31,32], and six articles compared the effect of the same FRCs on microleakage [9,10,11,33,34,35].

In total, eight out of nine included studies evaluated the effect of FRCs on fracture resistance using endodontically treated teeth, while only one was performed using non-endodontically treated teeth [4]. Four out of nine articles concluded that reinforcement with both FRC types could increase the fracture resistance of the restoration in the same manner without statistically significant differences [26,28,29,32]. Another four studies showed superior fracture resistance restorations, reinforced by glass FRCs (everStick C&B, everX Posterior) over polyethylene (Ribbond) FRCs; the differences were statistically significant in all of them [8,27,30,31]. Only one study showed that Ribbond has statistically higher resistance to fracture, compared to everStick NET, when the latter was bucco-lingually used on the base of the cavity or on top of it [4].

Regarding the failure mode, six out of nine studies assessed the failure mode of the restorations and divided it into favourable/reparable or unfavourable/irreparable [4,8,27,28,29,32]. Considering the studies that evaluate the effect of FRCs on microleakage, all six included studies concluded that reinforcing restorations with both FRC types significantly reduced microleakage, as compared to restorations without fiber inserts. One study showed that the Ribbond fiber insert group exhibited a significantly lower reduction in microleakage when compared to the everStick NET fiber inserts [35]. Table 1 summarizes details of the included studies.

Risk of bias of the included studies is summarized in Table 2. Briefly, the vast majority of the studies were classified as having medium risk of bias (4–6 YES), while three studies were classified with high bias risk [26,29,30]. However, some parameters were missing in most of the studies: sample size calculation and operator’s blindness.

## 4. Discussion

Fiber-reinforced composites are widely used to reinforce restorations of structurally weakened teeth [36,37]. Type of fibers, their orientation, resin impregnation as well as adhesion between fibers and resin play a crucial role in their reinforcing ability [38]. The present review was conducted to analyze the comparative data available in the literature that tested the influence of different fiber reinforcement types on the effectiveness of posterior large composite restoration. Based on these data, the most significant parameters, which tested the reinforcing performance of FRCs, could be divided into different groups.

### 4.1. Fracture Resistance

The tooth structure left after cavity preparation is a decisive factor to determine its fracture strength [2]. To enhance the strength of the remaining tooth structure, different restorative materials have been introduced, and different methods to improve the properties of the conventional materials have been applied. The selection of the suitable material to compensate the lost tooth structure and support the remaining tissue is fundamental to achieve successful treatment [39].

Different types of FRCs are among the most important examples of the modified composites due to their tooth strengthening effect [40]. According to the findings of this review, superior fracture strength values resulted from restorations supported by FRCs compared to non-fiber-reinforced composite restorations, which contrasts the findings of Belli et al. [7,15]. Some comparative studies found that polyethylene and glass FRCs similarly increased the fracture strength of the restorations while, in other studies, certain differences in the fracture strength values existed between polyethylene and glass FRCs. According to Kemaloglu et al. and others [26,28,29,32], there was a significant increase in the fracture strength in groups reinforced with FRC restorations, compared to class II cavities, restored with composite restorations without fiber reinforcement or unrestored cavities; however, there were no differences between the two FRC groups. This was explained by the modifying effect of short multidirectional glass fibers in SFRC or the multidirectional yarns and locked interwoven series of polyethylene fibers on the interfacial stresses, creating multitude paths of load [22,41]. This in turn helps in the redistribution of the occlusal forces and crack twisting, which reduce the stress intensity and prevent the rapid growth of the cracks. Moreover, composite restorations without fiber reinforcement also lack adequate fracture toughness, which is significantly lower than that of restorations with FRCs, and they have weak crack-arresting ability; therefore, they easily accelerate cracks. This could intensify the stresses at the crack-filler interface, which explains the catastrophic failure of the plain composite restorations and the reparable failures of FRC restorations resulted in these studies [28,32].

Sáry et al. have compared the fracture resistance of restorations reinforced with polyethylene (Ribbond) and two types of glass FRCs (everX Posterior and everStick NET) applied using different restorative techniques [4]. The results showed that restorations reinforced with Ribbond FRCs have statistically higher resistance to fracture, compared to restorations with everStick NET FRCs, when the latter was bucco-lingually used on the base of the cavity or on top of it. This could be attributed to the difference in the quantity (fiber volume) and the means of application of the used fibers. On the contrary, there were no statistical differences between restorations supported by Ribbond and the other restorations, when everX Posterior was used alone or in combination with everStick NET as an occlusal splint or circumferentially inside the cavity. The results indicate that the position of the bidirectional glass fiber net greatly affects its efficacy. The authors assumed that, when everX Posterior is used as a dentine substitution, the randomly oriented fibers exhibited an isotropic reinforcement effect in multiple directions, instead of in only a few specific directions [42]. In this study, cavities restored with everX Posterior are characterized by the highest percentage of favorable fractures; this is in accordance with a previous study by Frater et al., where the SFRC showed the ability to shift the fracture mode to favorable fracture [40]. This is mostly due to the ability of the SFRC substructure to support the overlying composite restoration and acts as a crack-prevention layer [43,44].

Previous studies, another three of which are included in this review, compared the fracture resistance of endodontically treated teeth with MOD, MO, and class I cavities, restored with SFRC (evevX Posterior), polyethylene FRC (Ribbond) or composites without fiber reinforcement [8,27,31]. Interestingly, one of these studies by Garlapati et al., showed that the fracture resistance of FRC restorations was even higher than the intact teeth [8]. Moreover, statistically significant results showed superior fracture resistance of everX Posterior over Ribbond FRC restorations and composite restorations without fiber reinforcement. According to the authors, the superior properties of the glass FRCs were mostly attributed to the composition, length, and distribution of the short glass fibers. everX Posterior consists of a combination of a resin matrix, randomly oriented E-glass fibers, and inorganic particulate fillers. The resin matrix comprises bis-GMA and TEGDMA cross-linked monomers, with linear PMMA. This unique resin combination allows for the formation of semi-IPN during the polymerization face; this results in improved bonding properties and toughness of the resin composite [22,45]. Regarding fiber length, the E-glass with bis-GMA has a critical fiber length between 0.5 and 1.6 mm and the short fibers present in everX posterior are equal to or greater than this length; this feature enables uniform stress distribution [46]. Moreover, these fibers can control polymerization shrinkage and marginal microleakage because of their fiber orientation [43].

Khan et al. compared the fracture resistance of root-canal-treated teeth restored with polyethylene (Ribbond) or glass (everStick C&B) FRCs under conventional composites [30]. Restorations reinforced with everStick C&B showed higher fracture strength values compared to restorations with Ribbond FRCs and composite restorations without fiber reinforcement, the differences were statistically significant. This was also explained by the presence of the semi-IPN, which enhances chemical bonding with the covering conventional composite. The authors also stated that the manual impregnation of Ribbond fibers could be inappropriately performed, which could create voids in the matrix and lead to the premature failure of the restoration [30]. These finding are in accordance with the findings of another study by Foek et al., in which the authors found that the adhesion of resin composite to polyethylene FRC was less favorable because of the difficulty in plasma coating and the impregnation of the polyethylene fibers [47]. Moreover, previous studies by Vallittu, using scanning electron microscopy, demonstrated a relatively poor fiber-matrix coupling, which negatively affected the resulting fracture toughness of polyethylene FRC [13].

All of the previous data were gained and compared, based on in vitro studies under static loading conditions, which vary from the clinical situation with dynamic loading conditions. Moreover, it is quite inequitable to compare the fracture resistance data of the laboratory studies, due to different varieties, such as the situations of the used teeth, the procedures of applying the restorations and the different study protocols; these could be considered as limitations of the included studies in the present review.

### 4.2. Microleakage and Marginal Adaptation

Microleakage is considered as one of the major limitations of composite restorations. This phenomenon happened because of the stresses generated at the restoration-tooth interface due to different causes such as polymerization shrinkage; repetitive fatigue cycles due to masticatory forces; fluctuation of the temperature in the oral cavity [10]. Placing a layer of FRCs within the cavity to reduce the composite polymerization shrinkage and microleakage is one of the most widely tested methods. The overall analysis in this review showed that using FRCs could effectively reduce microleakage around the restoration, compared to restorations without fibers.

Belli et al., and Basavanna et al., evaluated the microleakage of class II composite restorations when cavities were lined with polyethylene (Ribbond) or glass (everStick NET) FRCs in combination with flowable composite, and cavity margins in enamel, dentine or root surfaces. The authors stated that both tested FRCs similarly help to reduce occlusal leakage in Class II cavities with enamel and root margins [9,33]. The same findings were obtained by El. Mowafy et al., and Ahmed et al., who evaluated the effect of glass (everStick POST) and polyethylene (Ribbond) FRCs on the microleakage of Class II and Class V composite restorations, respectively, with gingival margins on root surfaces. The authors explained how the insertion of fibers into the resin composite causes a decrease in the composite mass, and less resin composite mass indicates less volumetric shrinkage because of the presence of a smaller organic matrix, and subsequently less microleakage. Moreover, the fibers help the first resin composite to resist pull-away from the edges toward curing light [10,34].

According to Ozel and Soyman, who compared the effect of everStick NET and Ribbond FRCs on microleakage and polymerization shrinkage, both types could decrease the microleakage scores in MOD cavities. However, the results of this study stated that the volumetric polymerization shrinkage of restoration reinforced with glass FRCs was lower than that of polyethylene FRCs, although the results were not statistically significant [11]. On the other hand, statistically significant differences between glass and polyethylene FRCs were obtained by Kumar et al. When the microleakage of class II composite restoration was tested, higher microleakage values were obtained in restorations reinforced with polyethylene FRC groups compared to glass FRC groups [35]. These findings are consistent with those of Kolbeck et al., in which the authors attributed the results to the difficulty in obtaining good adhesion between the manually impregnated polyethylene fibers and resin matrix, which is opposite to the pre-impregnated glass fibers by manufacturers [48].

It is important to highlight that all of the previously mentioned results were obtained from short-term laboratory studies. Different results could be obtained if long-term studies were performed.

## 5. Conclusions

Within the limitations of the present review, evidence from in vitro studies generally showed that FRCs tend to strengthen the restoration of structurally compromised teeth and improve their fracture resistance compared to composite restorations without fiber reinforcement. However, in almost all of the reviewed studies, short or continuous glass FRCs showed either the same performance or they exhibited better results than the polyethylene (woven) FRCs with regard to the fracture resistance. Moreover, using FRCs reduces the microleakage of restorations regardless of the fiber type.

## Figures and Tables

**Table 1:** Details of the included studies.

Study	Tested Parameter	Control Group	Type of Compared FRCs	FRCs Application Technique	Main Conclusion
Kmaloglu [28]	Fracture strength	MOD cavities of ETT restored with composite with no fiber reinforcement	Ribbond (Ribbond Inc., Seattle, WA, USA) and everX Posterior (GC Europe, Leuven, Belgium)	Ribbond was placed on the cavity floor BL; everX Posterior applied using bulk-fill (3 mm) technique.	Fiber reinforcement increased the fracture strength of teeth with large MOD cavities endodontically treated when compared to bulk-fill and nano-hybrid resin composites.
Ozsevik [31]	Fracture resistance	MOD cavities of ETT restored with composite with no fiber reinforcements	Ribbond and everX Posterior	Ribbond was placed on the cavity floor BL; everX Posterior applied using bulk-fill technique.	Using everX posterior under composite restorations resulted in fracture resistance similar to that of intact teeth. Furthermore, it reinforced root-filled teeth more than composite alone and ribbon and composite restorations.
Tekçe [29]	Fracture strength	No control	Ribbond and everX Posterior	Ribbond was placed on the cavity floor BL; everX Posterior applied using bulk-fill technique.	The polyethylene ribbon fibre-reinforced composite groups displayed similar fracture strength results with those of the short fibre-reinforced composite everX Posterior group.
Garlapati [8]	Fracture resistance	MOD cavities of ETT restored with composite with no fiber reinforcements	Ribbond and everX Posterior	Ribbond was placed on the cavity floor BL; everX Posterior applied using incremental technique.	Among the materials tested, endodontically treated teeth restored with everX posterior fiber reinforced composite showed superior fracture resistance.
Hiremath [26]	Fracture resistance	No control	Ribbond and everX Posterior	Ribbond was placed circumferentially against the entire inner surfaces; everX Posterior applied using bulk-fill technique.	Both FRC and polyethylene fibers (Ribbond) could be considered as an alternate to crown coverage, considering the insignificant difference in the values of fracture resistance when compared to that of natural tooth.
Khan [30]	Fracture resistance	MOD cavity of ETT restored with composite with no fiber reinforcements	Ribbond, everStick C&B (Stick Tech, GC member, Turku, Finland), Dentapreg (UFM, ADM AS, Brno, Czech Republic), and Bioctris fibers (Bio Composants Medicaux, Tullins, France)	Ribbond was placed on the cavity floor BL; two pieces of everStick C&B coated the cavity surface; Dentapreg coated the cavity surface; Bioctris coated the cavity surface.	Among the different fibers tested, Everstick and Bioctris demonstrated the highest fracture resistance. Thus, it can be inferred that E-glass system is able to reinforce teeth better than S2 glass or Polyethylene fibers.
Sah [27]	Fracture resistance	MOD cavities of ETT restored with composite with no fiber reinforcements	Ribbond and everX Posterior	Ribbond was placed on the cavity floor BL; everX Posterior applied using bulk-fill (3 mm) technique.	The mean load to fracture was highest for EverX posterior followed by Ribbond and Conventional Composite for different cavity configuration.
Sáry [4]	Fracture resistance	MOD cavities restored with composite with no fiber reinforcements	Ribbond, everX Posterior, and everStick NET (Stick Tech, GC member, Turku, Finland)	Ribbond was placed either on the base BL, on the top, as an occlusal splint, circumferentially or transcoronaly; everX Posterior applied using bulk-fill technique; everStick NET was applied with everX Posterior either on the cavity base BL, on the top BL, as an occlusal splint or circumferentially inside the cavity.	Incorporating polyethylene or a combination of short and bidirectional glass fibres in certain positions in direct restorations seems to be able to restore the fracture resistance of sound molar teeth.
Shah [32]	Fracture resistance	Cavities of ETT restored with composite with no fiber reinforcements	Ribbond and everX Posterior	Ribbond was placed on the cavity floor BL; everX Posterior applied using incremental technique.	Fibre reinforced composites when used in different cavity configurations of endodontically treated premolar yielded similar results.
Belli [9]	Microleakage	Class II cavities restored with composite with no fiber reinforcement	Ribbond and everX Posterior	Ribbond was placed on the cavity floor BL; everStick NET was placed on cavity floor	The use of flowable composite alone or in combination with polyethylene or glass fibers helps reduce occlusal leakage in class II adhesive cavities with enamel margins.
El. Mowafy [10]	Microleakage	Class II cavities restored with composite with no fiber reinforcement	Ribbond and everStick Post 0.9 mm (Stick Tech oy, GC member, Turku, Finland)	Ribbond was placed on the gingival floor; everStick Post was placed on the gingival floor.	The use of fiber inserts significantly reduced microleakage in Class II resin composite restorations with gingival margins on the root surface.
Ozel [11]	Microleakage and polymerization shrinkage	Class II cavities restored with composite with no fiber reinforcements	Ribbond and everStick NET	Ribbond was applied on the gingival and axial wall; everStick NET was applied on the gingival and axial wall.	Fiber nets in general decreased both microleakage and polymerization shrinkage.
Basavanna [33]	Microleakage	Class II cavities restored with composite with no fiber reinforcements	Ribbond and everStick NET	Ribbond was placed on the gingival floor; everStick NET was placed on the gingival floor.	The use of fiber inserts significantly reduces microleakage in class II resin composite restorationswith gigngival margins on the root surface, with no significant difference between the different fiber inserts groups.
Ahmed [34]	Microleakage	Class V cavities restored with composite with no fiber reinforcements	Ribbond and everStick Post 0.9 mm	Ribbond was positioned into the restoration at the gingival seat after polymerization of the first increment and before the application of the second increment; everStick Post was positioned as previously done with Ribbond.	Class V resin composite restorations bonded with a total etch adhesive had a significant reduction in mean microleakage scores when glass or polyethylene fibers were placed at the gingival cavo-surface margin
Kumar [35]	Microleakage	Class II cavities restored with composite with no fiber reinforcements	Ribbond and everStick NET	Ribbond was placed on the gingival floor; everStick NET was placed on the gingival floor.	Polyethylene fiber inserts group exhibited less reduction in microleakage when compared to Glass fiber inserts and Prepolymerized Composite fiber inserts.

ETT: endodontically treated teeth; MOD: mesio-occlusal-distal; BL: bucco-lingually.

**Table 2:** Risk of bias assessment.

Studies Evaluated	Control Group	Standardized Samples	Randomized Samples	Single Operator	Manufacture’s Instructions	Operator Blindness	Sample Size Calculation	Failure-mode Evaluation	Risk of Bias
Kemaloglu [28]	YES	YES	YES	NO	YES	NO	NO	YES	Medium
Ozsevik [31]	YES	YES	YES	NO	YES	NO	YES	NO	Medium
Tekce [29]	NO	YES	NO	NO	YES	NO	NO	YES	High
Garlapati [8]	YES	YES	YES	NO	YES	NO	NO	YES	Medium
Hiremath [26]	NO	YES	YES	NO	NO	NO	NO	NO	High
Khan [30]	YES	YES	NO	NO	YES	NO	NO	NO	High
Sah [27]	YES	YES	YES	NO	YES	NO	NO	YES	Medium
Sáry [4]	YES	YES	YES	YES	YES	NO	NO	YES	Medium
Shah [32]	YES	YES	NO	NO	YES	NO	NO	YES	Medium
Belli [9]	YES	YES	YES	NO	YES	YES	NO	NA	Medium
El. Mowafy [10]	YES	YES	YES	YES	NO	NO	NO	NA	Medium
Ozel [11]	YES	YES	YES	YES	YES	NO	NO	NA	Medium
Basavanna [33]	YES	YES	YES	NO	YES	NO	NO	NA	Medium
Ahmed [34]	YES	YES	YES	YES	YES	NO	NO	NA	Medium
Kumar [35]	YES	YES	YES	NO	YES	NO	NO	NA	Medium

NA: not applicable.

## Data Availability

Not applicable.
